# 

**Published:** 1885-12

**Authors:** 


DECEMBER, 1885.

Vol. III. No. 10.

### THE BRISTOL MEDICO-CHIRURGICAL JOURNAL

A Quarterly Journal of the Medical Sciences for the West of England and South Wales

*PUBLISHED UNDER THE AUSPICES OF*

*THE BRISTOL MEDICO-CHIRURGICAL SOCIETY.*

EDITED BY

J. GREIG SMITH,

M.A., F.R.S.E.

---

"Scire est nescire, nisi id me

Scire alius sciret."

---

BRISTOL: J. W. ARROWSMITH;
LONDON: J. & A. CHURCHILL, 11 NEW BURLINGTON STREET.

*Price One Shilling and Sixpence.*

